# Sex differences in the role of atypical PKC within the basolateral nucleus of the amygdala in a mouse hyperalgesic priming model

**DOI:** 10.1016/j.ynpai.2020.100049

**Published:** 2020-06-04

**Authors:** Daniela Baptista-de-Souza, Diana Tavares-Ferreira, Salim Megat, Ishwarya Sankaranarayanan, Stephanie Shiers, Christopher M. Flores, Sourav Ghosh, Ricardo Luiz Nunes-de-Souza, Azair Canto-de-Souza, Theodore J. Price

**Affiliations:** a Dept. Psychology, Federal University of Sao Carlos-UFSCar, Sao Carlos, SP 13565-905, Brazil; b University of Texas at Dallas, School of Behavioral and Brain Sciences and Center for Advanced Pain Studies, United States; c Janssen Research & Development, Neuroscience Therapeutic Area, San Diego, CA, United States; d Yale University School of Medicine, Department of Neurology, United States; e Joint Graduate Program in Physiological Sciences UFSCar/UNESP, São Carlos, SP 13565-905, Brazil; f Graduate Program in Psychology UFSCar, São Carlos, SP 13565-905, Brazil; g Lab. Pharmacology, School of Pharmaceutical Sciences, Univ. Estadual Paulista – UNESP, Araraquara, SP 14800-903, Brazil

**Keywords:** Sex differences, Hyperalgesic priming, Basolateral amygdala, ZIP, aPKC, GluA2

## Abstract

Though sex differences in chronic pain have been consistently described in the literature, their underlying neural mechanisms are poorly understood. Previous work in humans has demonstrated that men and women differentially invoke distinct brain regions and circuits in coping with subjective pain unpleasantness. The goal of the present work was to elucidate the molecular mechanisms in the basolateral nucleus of the amygdala (BLA) that modulate hyperalgesic priming, a pain plasticity model, in males and females. We used plantar incision as the first, priming stimulus and prostaglandin E_2_ (PGE_2_) as the second stimulus. We sought to assess whether hyperalgesic priming can be prevented or reversed by pharmacologically manipulating molecular targets in the BLA of male or female mice. We found that administering ZIP, a cell-permeable inhibitor of aPKC, into the BLA attenuated aspects of hyperalgesic priming induced by plantar incision in males and females. However, incision only upregulated PKCζ/PKMζ immunoreactivity in the BLA of male mice, and deficits in hyperalgesic priming were seen only when we restricted our analysis to male *Prkcz^−/−^* mice. On the other hand, intra-BLA microinjections of pep2m, a peptide that interferes with the trafficking and function of GluA2-containing AMPA receptors, a downstream target of aPKC, reduced mechanical hypersensitivity after plantar incision and disrupted the development of hyperalgesic priming in both male and female mice. In addition, pep2m treatment reduced facial grimacing and restored aberrant behavioral responses in the sucrose splash test in male and female primed mice. Immunofluorescence results demonstrated upregulation of GluA2 expression in the BLA of male and female primed mice, consistent with pep2m findings. We conclude that, in a model of incision-induced hyperalgesic priming, PKCζ/PKMζ in the BLA is critical for the development of hyperalgesic priming in males, while GluA2 in the BLA is crucial for the expression of both reflexive and affective pain-related behaviors in both male and female mice in this model. Our findings add to a growing body of evidence of sex differences in molecular pain mechanisms in the brain.

## Highlights

• Intra-BLA ZIP, a cell-permeable inhibitor of aPKC, attenuated hyperalgesic priming induced by plantar incision in both sexes.
• Priming upregulated aPKC in the BLA only in male mice.
• Deficits in hyperalgesic priming were only seen in male *Prkcz^−/−^* mice.
• Pep2m, a peptide that interferes GluA2 AMPA receptors, reduced priming in both male and female mice.
• GluA2 expression was upregulated in the BLA of male and female primed mice.
• GluA2 in the BLA is crucial for the initiation of both reflexive and affective pain-related behaviors in both sexes.

## 1 Introduction

Chronic pain is more prevalent in women than men (Andersson et al., 1993, Myers et al., 2003, Racine et al., 2012). Basic mechanisms that promote chronic pain in females are poorly understood in comparison to males, which is likely due to a preceding-bias for studying males in preclinical and clinical pain research (Mogil, 2012, Boerner et al., 2018). Recent NIH initiatives (Collins and Tabak, 2014) requiring mixed-sex animal testing have encouraged the discovery of sex-specific pain mechanisms that may underlie differences between males and females (Berkley, 1997, Craft et al., 2004, Aloisi and Bonifazi, 2006, Fillingim et al., 2009, Bartley and Fillingim, 2013, Mogil, 2016, Chillingworth et al., 2006, Vacca et al., 2014, Vacca et al., 2016, Rosen et al., 2019). One of the best examples identified to date is the dominant role of microglia in chronic nociceptive hypersensitivity in males (Taves et al., 2016). Recent work suggests these sex-specific mechanisms in the monocyte lineage may be preserved in human patients with neuropathic pain (North et al., 2019). Therefore, it is of critical importance to understand how chronic pain develops in females in order to develop effective pain therapeutics for women (Storesund et al., 2016, Chow et al., 2017).

The development of chronic pain can be initiated by acute tissue damage that sensitizes neurons, creating a primed state after the injury has healed and an exaggerated nociceptive response upon subsequent exposure to small doses of inflammatory mediators (Kandasamy and Price, 2015, Price and Inyang, 2015). Surgical incision can prime humans/animals to develop chronic pain/nociceptive hypersensitivity (Aasvang and Kehlet, 2005, Kehlet et al., 2006). The hyperalgesic priming model represents an important tool for investigating neuronal plasticity mechanisms that may be important in the development of chronic pain (Reichling and Levine, 2009). In hyperalgesic priming models a first stimulus is given, such as plantar incision, inflammatory mediators like carrageenan or interleukin 6 and animals are allowed to recover from this first stimulus. Animals are then challenged with a second stimulus, which is usually PGE_2_, although other mediators have been used. In primed animals this second stimulus causes a more intense response to the stimulus than in naïve animals and the duration of the response is also greatly increased. Interventions given around the time of the first stimulus, or before the second stimulus, can test mechanisms of initiation or maintenance of hyperalgesic priming, respectively [reviewed in (Kandasamy and Price, 2015)].

Hyperalgesic priming mechanisms are highly sexually dimorphic (Joseph et al., 2003, Khomula et al., 2017, Megat et al., 2018, Paige et al., 2018, Moy et al., 2019). Some of these nociceptive differences have been attributed to differences in ascending and/or descending pain transmission pathways (Niesters et al., 2010, Megat et al., 2018), but little research has investigated the molecular factors underlying these differences. Recent findings demonstrate that sex dimorphisms in pain responses may involve differences in emotion-processing centers (Girard-Tremblay et al., 2014, Strath RESaLJ, 2018). In this context, it was shown that men mobilize ventromedial prefrontal cortex areas that mediate threat-control circuits as an emotional component related to anxiety, whereas women recruit emotion-processing centers, such as the perigenual amygdaloid complex, to cope with pain unpleasantness (Girard-Tremblay et al., 2014).

Protein kinase M zeta (PKMζ) is an autonomously active atypical PKC (aPKC) isoform, which was recently implicated as having a male-specific role in the maintenance of chronic pain (Nasir et al., 2016, George et al., 2019, Martin et al., 2019). This protein may also be involved in the modulation of memory processes in several CNS areas, such as hippocampus, prefrontal cortex and amygdala (Evuarherhe et al., 2014, Sun and Alkon, 2014, Opendak et al., 2018), in particular the BLA (Bonini et al., 2005, Kwapis et al., 2012). The BLA modulates fear and anxiety, which are directly related to affective aspects of pain (Phelps and LeDoux, 2005, Neugebauer, 2015); and a neuronal ensemble that encodes pain unpleasantness has recently been identified in the BLA (Corder et al., 2019). The aim of the present study was to investigate the role of PKCζ/PKMζ in the BLA on evoked and affective pain behaviors in a rodent model of hyperalgesic priming. In addition, we also investigated the potential role of trafficking of AMPA receptors, because their trafficking is known to be regulated by aPKCs. We find a male specific role of PKCζ/PKMζ in the BLA in the hyperalgesic priming model. However, AMPA receptor changes occur in both male and female mice, and interference with AMPA receptor trafficking in the BLA interrupts priming in male and female mice. Therefore, our work reveals a new mechanism for hyperalgesic priming involving sexually dimorphic signaling in the BLA.

## 2 Materials and methods

### 2.1 Animals

Animals were housed on a 12 h light/dark cycle, with food and water available *ad libitum*. All procedures were performed during the light cycle. All experiments were approved by the Institutional Animal Care and Use Committee at the University of Texas at Dallas and conducted in accordance with the National Institutes of Health and International Association for the Study of Pain guidelines. Experimental C57BL6/J wild-type (WT) animals were obtained from an internally maintained C57BL/6J colony at the University of Texas at Dallas. *Prkcz^−/−^* mice were generated as described previously (Chibly et al., 2018). *Prkcz^−/−^* mice and wildtype littermates were used for those experiments. Twelve week-old male and female mice were used for all experiments. Mice were randomly assigned to groups by a blinded experimenter. Experimenters performing behavioral experiments were blinded to genotype and drug treatment.

### 2.2 Drugs

Myristoylated ZIP, pep2m, and scrambled peptide were acquired from Tocris Bioscience; prostaglandin E_2_ (PGE_2_) was obtained from Cayman Chemical Company; and nerve growth factor (NGF) was from R&D Systems. ZIP, pep2m and scrambled peptide stock solutions were prepared in sterile 0.9% saline and diluted from this stock solution for injection. PGE_2_ stock solutions were prepared in 100% ethanol and diluted in sterile saline for injection. Doses used in this study were based on our previous work (Asiedu et al., 2011) and other publications (Shi et al., 2001, Pastalkova et al., 2006, Shema et al., 2007). ZIP is not a selective inhibitor of aPKCs (Lee et al., 2013, Volk et al., 2013). Pep2m interferes with GluA2 binding to NSF and the effect of the peptide is absent in GluA2 KO mice, suggesting it is a specific tool peptide (Shi et al., 2001).

### 2.3 Incision surgery and hyperalgesic priming

A 5 mm longitudinal incision was made by using a number 11 blade through skin, fascia and muscle of the plantar aspect of the left hind paw in an anesthetized mouse (1% isoflurane). The skin was closed with 2–5 mm silk sutures (Banik et al., 2006). The sutures were removed after 48 hr. Animals were allowed to recover for 24 hr, and then paw withdrawal thresholds were measured at several time-points (1, 3, 11 and 14 days) post-incision. Mice whose mechanical thresholds returned to baseline following incision (at 11 or 14 days) received an intraplantar injection of PGE_2_ (100 ng/25 µl) as the second stimulus (Asiedu et al., 2011).

### 2.4 Stereotaxic surgery and microinjection

Stainless steel guided cannulas were implanted into the bilateral BLA [anterior/posterior, −1.2 mm; medial/lateral, 3.1 mm; dorsal/ventral, 4.6 mm] (Paxinos, 2001). ZIP, pep2m or scrambled peptide were microinjected into the bilateral BLA using a 33-gauge stainless steel needle that extended 2 mm beyond the tip of the guide cannula. The injector was connected to a 10 µl Hamilton microsyringe via polyethylene tubing (PE-10), and the rate of flow was controlled by an infusion pump programmed to deliver 0.1 µl of each solution over a period of 60 sec. The microinjection procedure consisted of gently restraining the mouse, inserting the injector, infusing the solution for 60 sec and keeping the injector in place for an additional 90 sec. The movement of a small air bubble in the PE-10 tubing, during and after the microinjection, confirmed the delivery of the solution. ZIP or scrambled peptide was given into the BLA only during the initiation phase (24 hr after plantar incision). Pep2m was microinjected during the initiation phase and prior to second stimulus (24 hr before PGE_2_ plantar injection). We gave intra-BLA injections 24 hr after incision because spontaneous nociceptor discharges continue for approximately 24 hrs after incision and coincide with spontaneous pain behaviors (Banik and Brennan, 2004). We reasoned that timing treatment with the cessation of spontaneous nociceptor discharges would offer the best opportunity for reversal of plasticity in brain circuits (for review see (Kandasamy and Price, 2015, Price and Inyang, 2015). We gave treatments only at the 24 hr after incision time point for ZIP because the tool compound likely acts via a mechanism that is distinct from aPKC, as shown in our results and in previous students cited herein. Therefore, we decided to do these experiments in the smallest number of animals possible. We used pep2m at 24 hrs after incision and at later time points because this tool is likely to act specifically on GluA2 trafficking, justifying additional animal experiments.

After each experiment, all animals received an intra-BLA infusion of Evans blue (1%) to verify the injection site. The mice were then euthanized, and their brains were placed into 10% formalin for at least 48 hr. Following fixation, the brains were cryoprotected in 30% sucrose, embedded in optimum cutting temperature (O.C.T.) media, sectioned coronally at 35 μm thickness on a cryostat and mounted onto charged-slides. BLA cannula placement was confirmed by light microscopy of Evan’s blue staining using the Paxinos and Franklin Mouse Brain atlas (Franklin and Paxions, 2012). Mice with injection sites outside the BLA were excluded from the study (supplementary Fig. 1).

### 2.5 Mechanical withdrawal threshold

Mice were habituated to transparent acrylic boxes with wire mesh floors for 1 hr before testing. Von Frey monofilaments (Stoelting) were firmly applied to the plantar surface of each hind paw for 0.5 sec. Withdrawal thresholds were calculated using the up-down method of Dixon (Chaplan et al., 1994). Mechanical withdrawal thresholds were assessed prior to surgery (baseline) and at multiple time points post-incision, as indicated in the graphs and figure captions. When von Frey testing was done on the same day as an injection in the BLA, the behavioral assessment was done 4–5 hrs following the drug injection.

### 2.6 Mouse grimace scale

The Mouse Grimace Scale (MGS) was used to quantify affective aspects of nociception (Langford et al., 2010). Facial expressions were scored using the facial action coding system at 24 and 48 hr after plantar incision and at 3 and 24 hr after intraplantar injection of PGE_2_. When grimace testing was done on the same day as an injection in the BLA, the behavioral assessment was done 4–5 hrs following the drug injection.

### 2.7 Sucrose splash test

10% sucrose solution was applied to the dorsal coat of the mouse. After application, the latency and frequency of grooming was recorded for 5 min as an index of self-care and motivational behavior (Isingrini et al., 2010). The Sucrose splash test was performed 24 hr after PGE_2_ injections.

### 2.8 Immunohistochemistry

Mice (4 to 5 per group) were anesthetized by the use of a mixture of ketamine (80 mg/kg) and xylazine (12 mg/kg) and transcardially perfused with 30 mL of 1X phosphate buffered saline (PBS), at pH 7.4 followed by 50 mL of fresh 4% formalin. The brain was dissected and transferred to a 30% sucrose solution in PBS for 48 hr at 4 °C. After the brains had submerged, the tissues were embedded in OCT and sectioned at 35 μm thickness on a cryostat. Sections were placed in serial order in a 12-well plate containing 0.1 M phosphate buffer (PB) with 0.01% sodium azide. Sections were washed 3 times in 0.1 M PB and then incubated in a blocking solution, containing 10% Normal Goat Serum and 0.3% Triton-X 100 in 0.1 M PB, for 1 h at room temperature with gentle rocking. Sections were incubated for 3 hr at room temperature with primary antibody that was diluted in blocking solution. The primary antibodies used were: rabbit-anti-PKCζ (Abcam, #108970; 1:1000 working dilution) and mouse-anti-GluA2 (Thermo Fisher Scientific, # 32-0300 – 1:1000 working concentration). The anti-PKCζ antibody was validated for specificity using a cell line (HAP1) where the PKCζ gene was deleted (details given on Abcam website). The GluA2 antibody was validated in previously published studies (Moga et al., 2003, Vazquez-Sanroman et al., 2015). Sections were washed 5 times in 0.1 M PB and then incubated for 1 hr at room temperature with secondary antibody (1:2000 each) diluted in blocking solution. The secondary antibodies used were: goat-anti-rabbit 488H&L (Thermo Fisher Scientific, #A21429), and goat-anti-mouse 488 IgG2a (Thermo Fisher Scientific, #A21131). Following secondary incubation, sections were washed 5 times in 0.1 M PB, mounted onto glass slides, cover-slipped using Prolong Gold Antifade (Thermo Fisher Scientific, #P36930) and sealed with nail polish once cured. The images of four BLA slices from each animal were acquired using a Olympus FV1200 confocal microscope, and images were analyzed using ImageJ software (NIH). Background staining was subtracted from each image prior to image analysis (but background was not removed from images shown in the paper). The corrected total cellular fluorescence (CTCF = Integrated Density − [Area of selected tissue area × Mean fluorescence of background readings], done as described previously (Burgess et al., 2010, McCloy et al., 2014)) of the entire bilateral amygdaloid nucleus was measured by subtracting the background fluorescence from the integrated intensity.

### 2.9 Data analysis and statistics

All data are represented as mean ± SEM. All analyses were performed using GraphPad Prism v6.0. Single comparisons were performed using Student’s *t-*test, and multiple comparisons were performed using a two-way ANOVA with Bonferroni *post hoc* tests for group comparisons.

## 3 Results

### 3.1 Deficit in hyperalgesic priming in male *Prkcz^−/−^* mice

**Fig. 1:**
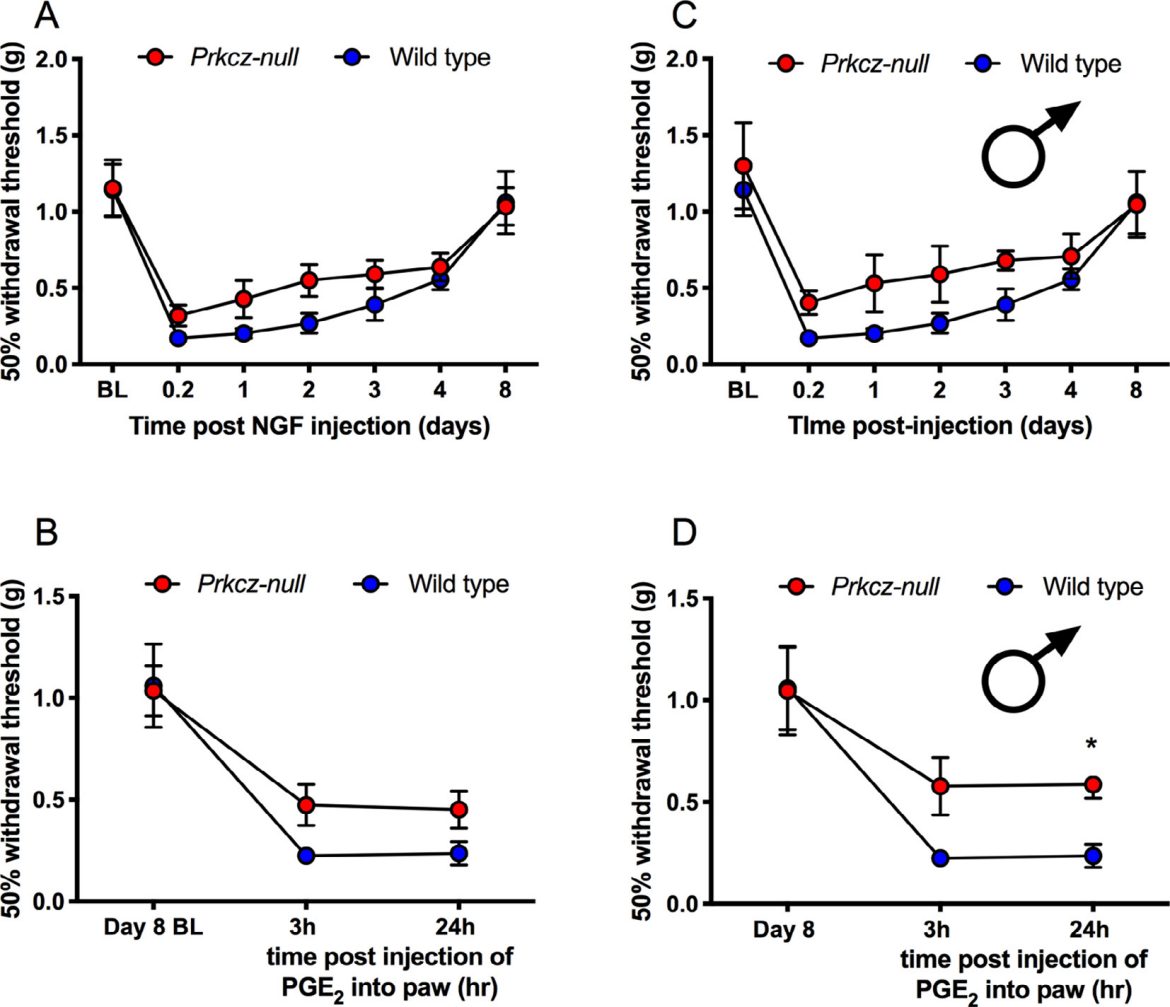
*Decreased hyperalgesic priming in male Prkcz^−/−^ mice.***(A)** Male and female *Prkcz^−^*^/^*^−^* and wild type mouse responses to von Frey stimulation in response to NGF (50 ng) treatment. **(B)** Male and female *Prkcz^−^*^/^*^−^* and wild type mouse responses to PGE_2_ (100 ng) after NGF priming. **(C)** Male *Prkcz^−^*^/^*^−^* and wild type mouse responses to von Frey stimulation in response to NGF (50 ng) treatment. **(D)** Male *Prkcz^−^*^/^*^−^* and wild type mouse responses to PGE_2_ (100 ng) after NGF priming. Two-way ANOVA with Bonferroni *post hoc* test *p < 0.05. WT n = 5 males per group; *Prkcz^−^*^/^*^−^* males n = 3; *Prkcz^−^*^/^*^−^* females n = 2.

Previous studies using *Prkcz^−/−^* mice have demonstrated a sex-specific role for PKCζ/PKMζ in preclinical models that are similar to hyperalgesic priming (Nasir et al., 2016, George et al., 2019). We used an independently-generated *Prkcz^−/−^* mouse (Chibly et al., 2018) to assess a potential sex-specific role of PKCζ/PKMζ in hyperalgesic priming using NGF as the priming stimulus. *Prkcz^−/−^* mice bred slowly and had small litters, so a limited number of mice were generated for these experiments. When both sexes were considered together, there was no difference in NGF-induced mechanical hypersensitivity (Fig. 1A) or the response to the second stimulus, PGE_2_ (Fig. 1B), between genotypes. However, when considering males alone, while there was similarly no difference between genotypes in NGF-induced mechanical hypersensitivity, there was a statistically significant difference in the response to the second stimulus (Fig. 1C and D). This finding is consistent with previous studies using a different *Prkcz^−/−^* mouse line and is also in line with pharmacological manipulation of aPKC in the spinal cord wherein an effect is only seen in male rodents (Nasir et al., 2016, George et al., 2019, Martin et al., 2019).

### 3.2 aPKC inhibition within the BLA influences incision-evoked nociception

**Fig. 2:**
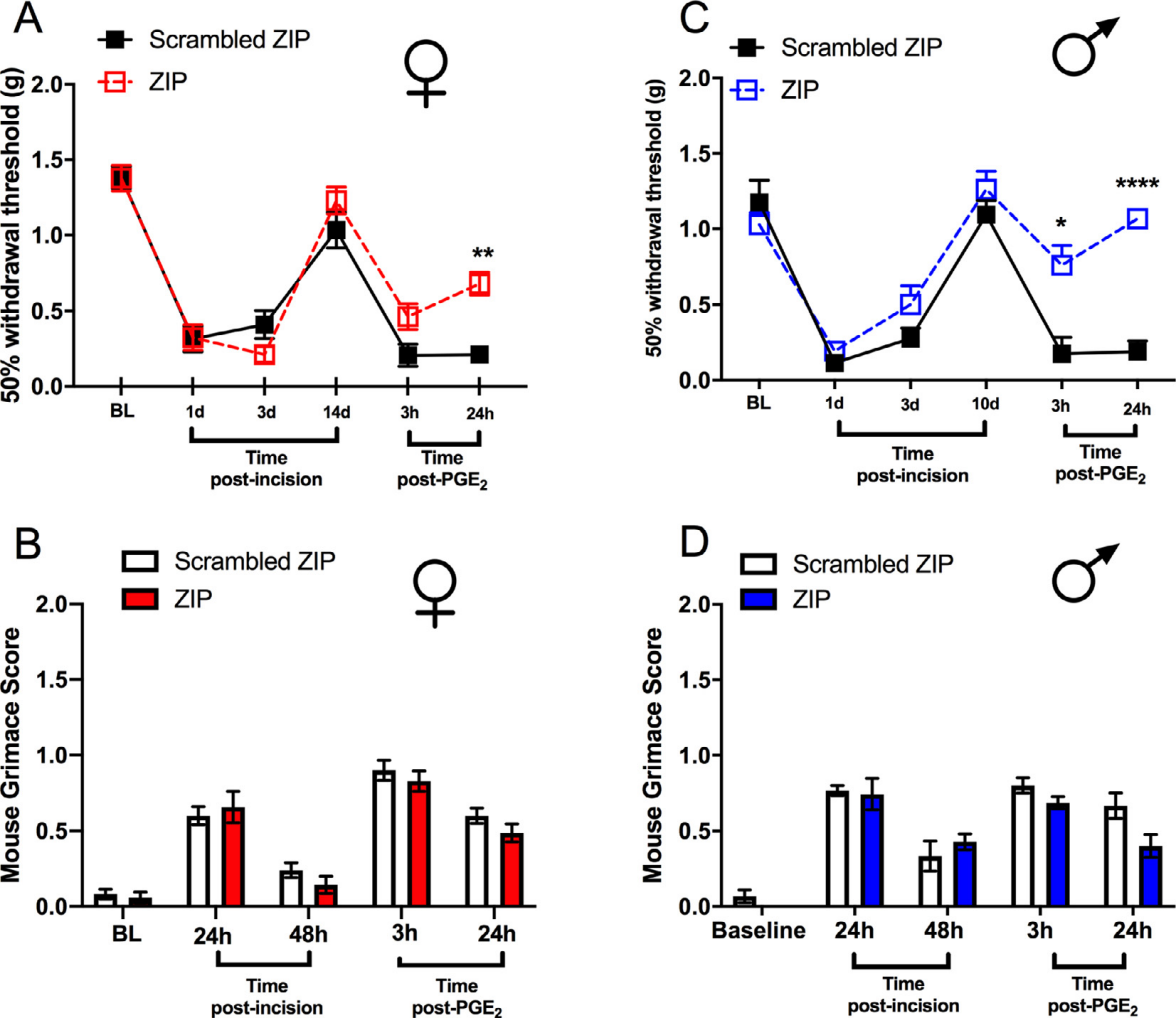
*Effects of bilateral intra-BLA microinjections of ZIP (*10 µ*g/ml) in males and females subjected to a plantar incision.* Effects of ZIP and scrambled control peptide given into the BLA 24 hr after incision in female mice in the von Frey test **(A)** and on the mouse grimace scale **(B).** Effects of ZIP and scrambled control peptide given into the BLA 24 hr after incision in male mice in the von Frey test **(C)** and on the mouse grimace scale **(D)**. Two-way ANOVA with Bonferroni *post hoc* test *p < 0.05; **p < 0.01; ***p < 0.001. Female groups n = 7 (ZIP); n = 10 (scrambled). Male groups n = 7 (ZIP); n = 6 (scrambled).

Atypical PKC has been implicated in nociception at the level of the spinal cord, and in higher brain centers, such as the anterior cingulate cortex (ACC) (Li et al., 2010, Asiedu et al., 2011, Laferriere et al., 2011, King et al., 2012, Melemedjian et al., 2013, Price and Ghosh, 2013, Nasir et al., 2016). Neuronal plasticity is induced in the BLA in many chronic pain models (Neugebauer, 2015), but a potential role for aPKCs in this brain site have not been tested in pain models. We used incisional surgery and hyperalgesic priming following this injury with intra-BLA infusion of ZIP to assess a potential role for aPKCs in post-surgical nociceptive hypersensitivity and hyperalgesic priming in male and female mice. We chose the incisional model because of its clinical relevance to the development of chronic pain after surgery (Kehlet et al., 2006). Male and female WT mice received an intra-BLA microinjection of ZIP or scrambled peptide 1 day following plantar incision. Microinjection of ZIP into the BLA had no effect on mechanical hypersensitivity or grimace responses following incision in either sex (Fig. 2A-D). However, when we examined the response to the second stimulus, we noted a significant decrease in PGE_2_-evoked mechanical hypersensitivity in both male and female mice (Fig. 2A and C). There were no differences in grimace scores in either sex (Fig. 2B and D).

**Fig. 3:**
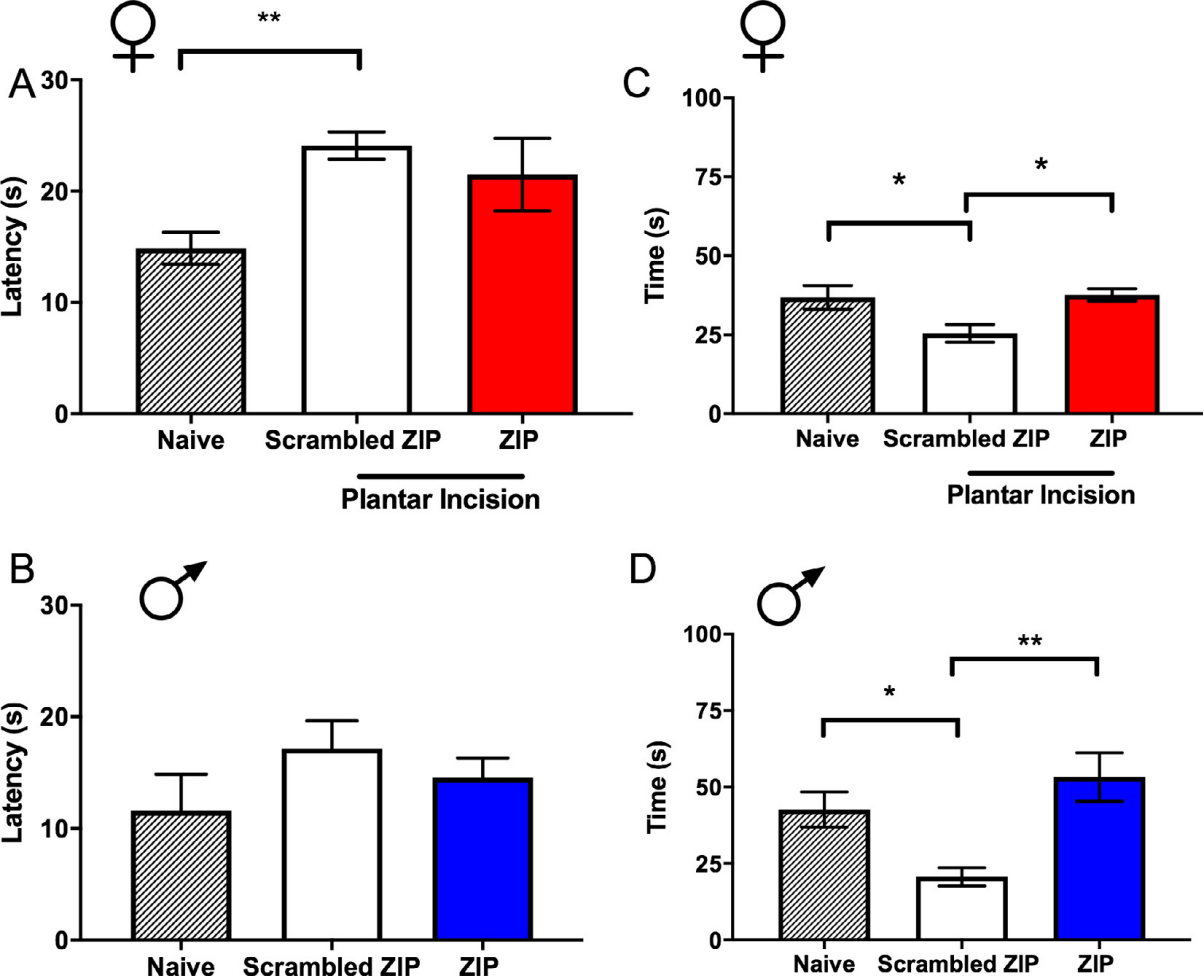
*Effects of bilateral intra-BLA microinjections of ZIP on sucrose splash test behaviors.* Effects of ZIP and scrambled control peptide given into the BLA 24 hr after incision in the sucrose splash test performed 24 hr after PGE_2_ injections. Incision and ZIP or scrambled peptide administration (10 μg/mL) effects on latency to begin grooming in female **(A)** and male mice **(B)**. Incision and ZIP or scrambled peptide administration (10 μg/mL) into the bilateral BLA effects on duration of grooming in female **(C)** and male mice **(D)**. One way ANOVA with Bonferroni *post hoc* test *p < 0.05; **p < 0.01. Female groups: naïve n = 8; n = 6 (ZIP); n = 9 (scrambled). Male groups: naïve n = 7; n = 7 (ZIP); n = 6 (scrambled).

We also assessed behaviors in the sucrose splash test in these mice. The latency to begin grooming was decreased in female mice who received incision and PGE_2_ treatment with intra-BLA scrambled peptide, but there was no change in the ZIP treated mice (Fig. 3A). No differences were seen in male mice for any treatment (Fig. 3B). A significant decrease in the total duration of grooming behaviors was observed in female (Fig. 3C) and male (Fig. 3D) mice given incision and treated with intra-BLA scrambled peptide. This effect was reversed in both sexes by intra-BLA ZIP treatment. These findings suggest the presence of a decreased motivational state in male and female mice that had previous incision surgery and that were exposed to the second stimulus. Like mechanical hypersensitivity in priming, this effect was reversed by pre-treatment with intra-BLA ZIP, in both sexes.

### 3.3 Changes in PKCζ/PKMζ expression in the BLA in incision-evoked hyperalgesic priming

**Fig. 4:**
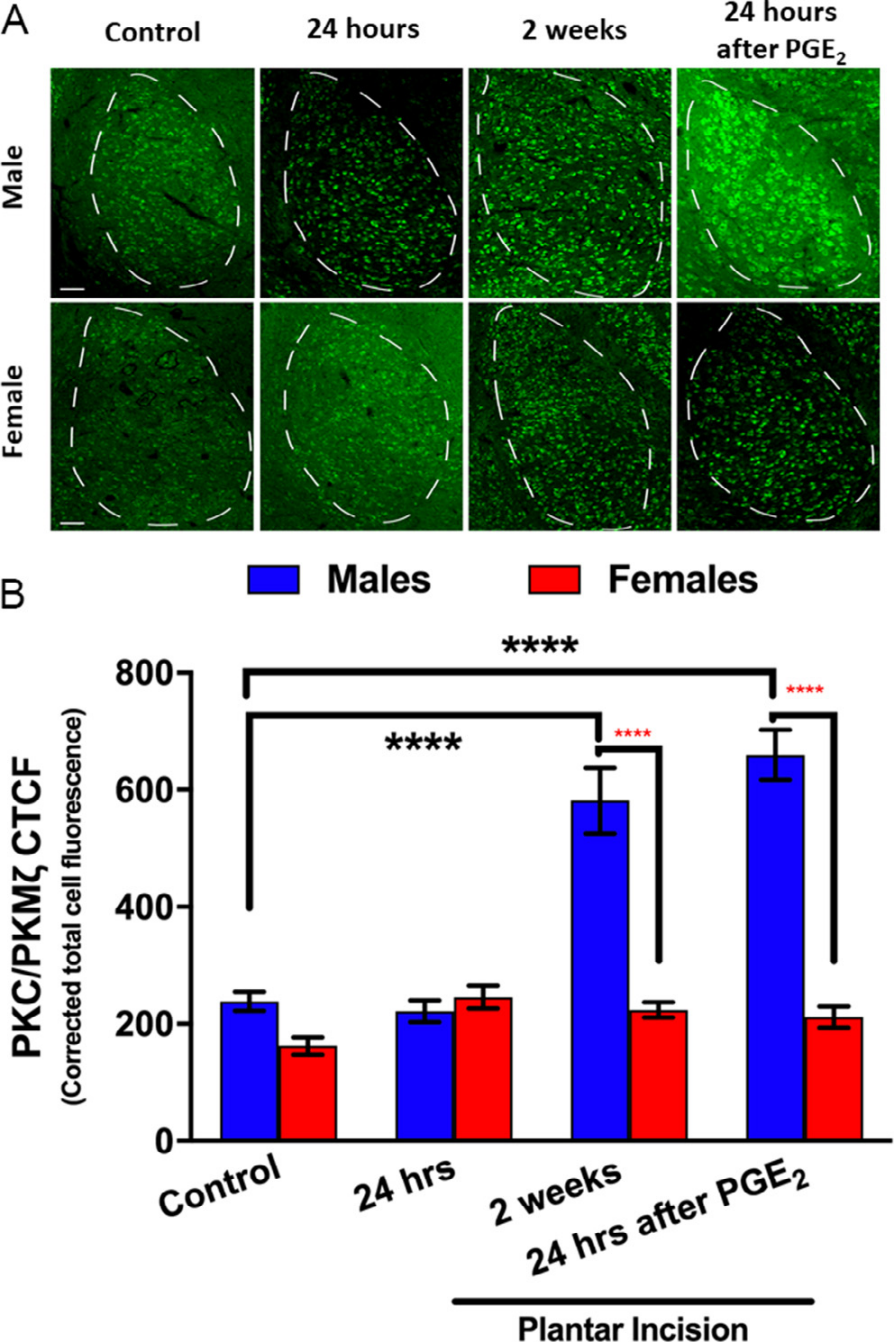
*Changes in PKCζ/PKMζ expression in BLA following hyperalgesic priming in male and female mice.***(A)** Representative 20X images showing PKCζ/PKMζ immunoreactivity in female and male mice without incision (control), 24 hr after incision, 2 weeks after incision (and before PGE_2_ treatment) and 24 hr after PGE_2_ injection in primed mice. **(B)** Corrected total cell fluorescence (CTCF) in males and females submitted to plantar incision (n = 4–5 group). ^#^p < 0.05 versus control group *p < 0.05 versus male mice group submitted to the same condition. Two-way ANOVA with Bonferroni *post hoc* test ****p < 0.0001. Black stars for difference versus control. Red stars for difference by sex. Scale bar = 50 μm. (For interpretation of the references to colour in this figure legend, the reader is referred to the web version of this article.)

Our genetic findings, showing an effect on hyperalgesic priming in males, and our pharmacological findings, showing an effect of intra-BLA ZIP in both sexes, are opposed to each other. A possible explanation is that ZIP has none specific effects in the BLA that reduce hyperalgesic priming in males and females. This idea is supported by the known non-specific pharmacology of ZIP (Lee et al., 2013, Volk et al., 2013). We examined whether changes in aPKC expression occur in the BLA over the course of our hyperalgesic priming experiments. We measured immunofluorescence for total PKCζ/PKMζ, which is known to increase during synaptic plasticity (Bonini et al., 2005, Sun and Alkon, 2014, Opendak et al., 2018). Males displayed higher PKCζ/PKMζ expression in the BLA at 2 weeks post-incision, just prior to the time of PGE_2_ injection, as well as after the PGE_2_ injection (Fig. 4A and B). No changes in PKCζ/PKMζ immunoreactivity were seen in females at any time point (Fig. 4A and B). Therefore, the most parsimonious explanation for our findings are that there is a male-specific role of the *Prkcz* gene in hyperalgesic priming, and the effects seen in females in our experiments are likely attributable to a different mechanism.

### 3.4 GluA2 trafficking in the BLA contributes to hyperalgesic priming in male and female mice

**Fig. 5:**
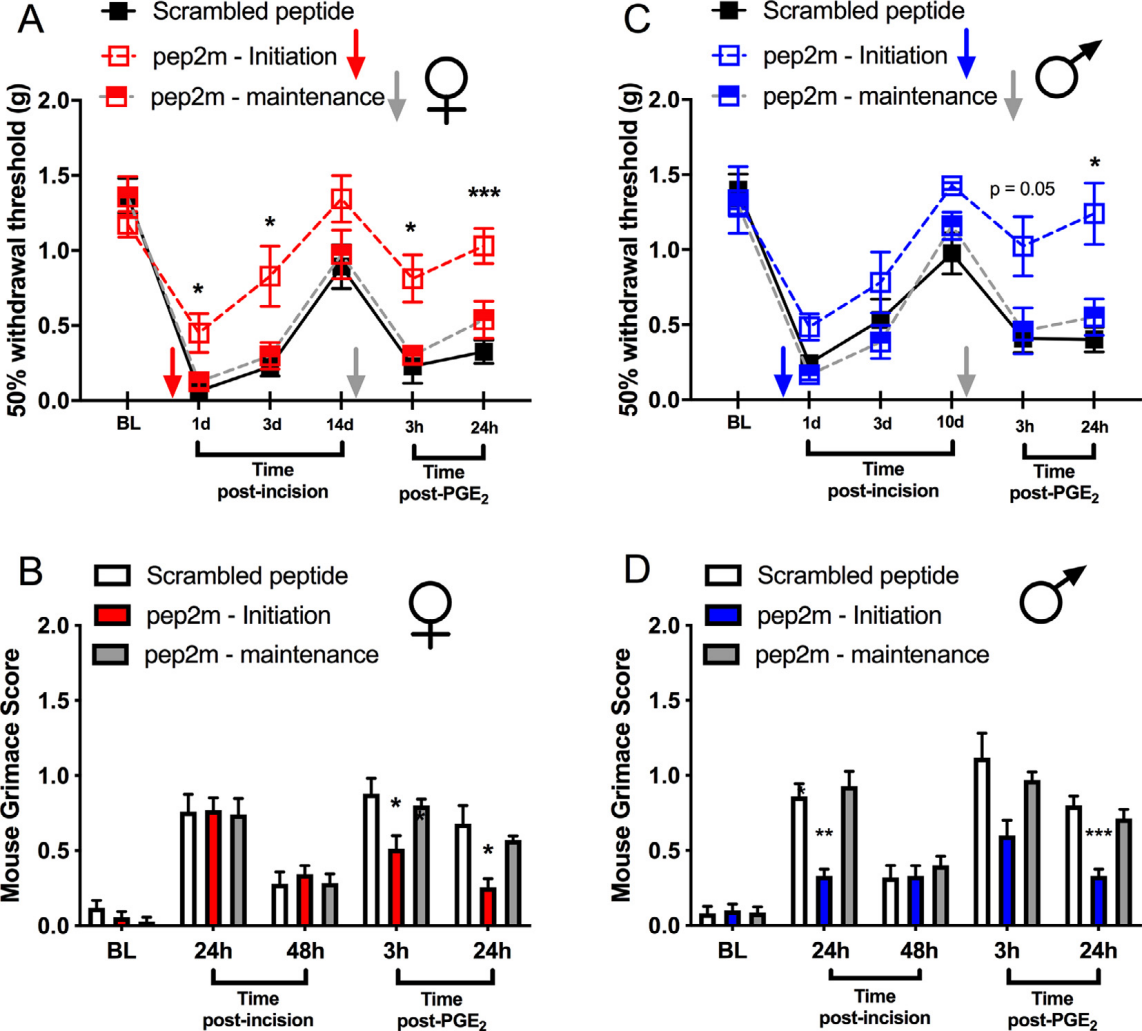
*Effects of bilateral intra-BLA microinjections of pep2m (*10 µ*g/ml) in males and females subjected to the plantar incision.* Effects of pep2m and scrambled control peptide given into the BLA 24 hr after incision (initiation) or 24 hr prior to PGE_2_ (maintenance) in female mice in the von Frey test **(A)** and on the mouse grimace scale **(B)**. Effects of pep2m and scrambled control peptide given into the BLA 24 hr after incision (initiation) or 24 hr prior to PGE_2_ in male mice in the von Frey test **(C)** and on the mouse grimace scale **(D)**. Two-way ANOVA with Bonferroni *post hoc* test *p < 0.05; **p < 0.01; ***p < 0.001; ****p < 0.0001. Female groups n = 7 (ZIP, initiation); n = 7 (ZIP, maintenance); n = 5 (scrambled). Male groups n = 6 (ZIP, initiation); n = 7 (ZIP, maintenance); n = 5 (scrambled).

To further explore mechanisms of hyperalgesic priming in the BLA in males and females, we performed microinjections of pep2m, a peptide that interferes with GluA2 trafficking and function, which has been described as a downstream target of aPKC (Migues et al., 2010), although this GluA2 mechanism does not show sex differences in pain models (George et al., 2019). We used two different experimental designs, in which intra-BLA microinjections of pep2m were administered either 24 hr after plantar incision during the initiation phase of priming or 24 hr before the PGE_2_ injections during the maintenance phase of priming. When pep2m was given 1 day following plantar incision, female mice showed a decrease in incision-evoked mechanical hypersensitivity and in hyperalgesic priming (Fig. 5A). Unlike with ZIP treatment, grimacing was also reduced in the priming phase with pep2m treatment in females 1 day following incision (Fig. 5B). No effect was seen when pep2m was given during the maintenance phase of hyperalgesic priming (Fig. 5A and B). Similar effects were seen in males with pep2m, except that there was a significant effect on grimacing behavior following incision with the initiation treatment paradigm (Fig. 5C and D). Again, in males, there was no effect of the pep2m treatment given into the BLA during the maintenance phase of hyperalgesic priming (Fig. 5C and D).

**Fig. 6:**
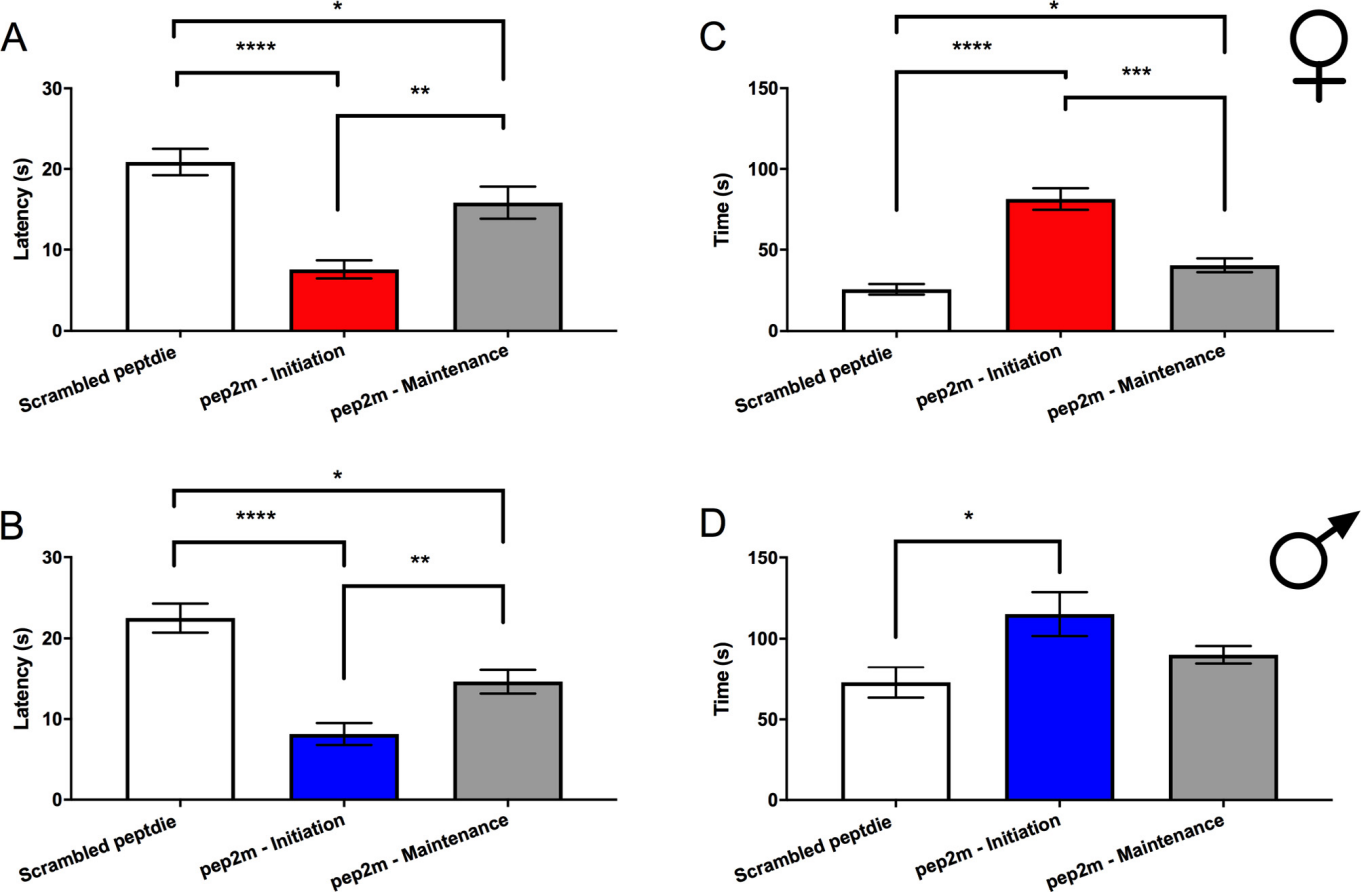
*Effects of bilateral intra-BLA microinjections of pep2m on sucrose splash test behaviors.* Effects of pep2m given at 24 hr after incision (initiation), or 24 hr prior to PGE_2_ (maintenance), or scrambled peptide administration (all 10 μg/mL) in sucrose splash test performed 24 hr after PGE_2_ injections. Incision and effects on latency to begin grooming in female **(A)** and male mice **(B)**. Treatment effects on duration of grooming in female **(C)** and male mice **(D)**. One way ANOVA with Bonferroni *post hoc* test *p < 0.05; **p < 0.01; ***p < 0.001; ****p < 0.0001. Female groups n = 7 (ZIP, initiation); n = 7 (ZIP, maintenance); n = 7 (scrambled). Male groups n = 6 (ZIP, initiation); n = 5 (ZIP, maintenance); n = 6 (scrambled).

Our results with the sucrose splash test (Fig. 3) show differences in observed behavior in primed mice. We tested for effects of intra-BLA infusion of pep2m in this test in mice receiving an incision and then treated with PGE_2_ as the second stimulus. We observed that intra-BLA pep2m decreased latency to grooming in male and female mice but only when given at the 24-hr time point after incision (Fig. 6A and B). Grooming time was greater in the mice treated with pep2m (both male and female), but again, only in mice treated at the 24-hr time point after incision (Fig. 6C and D). We conclude that disruption of GluA2 in the BLA during the initiation phase of hyperalgesic priming alters the behavioral response in primed mice in the sucrose splash test.

### 3.5 Changes in GluA2 expression in the BLA in incision-evoked hyperalgesic priming

**Fig. 7:**
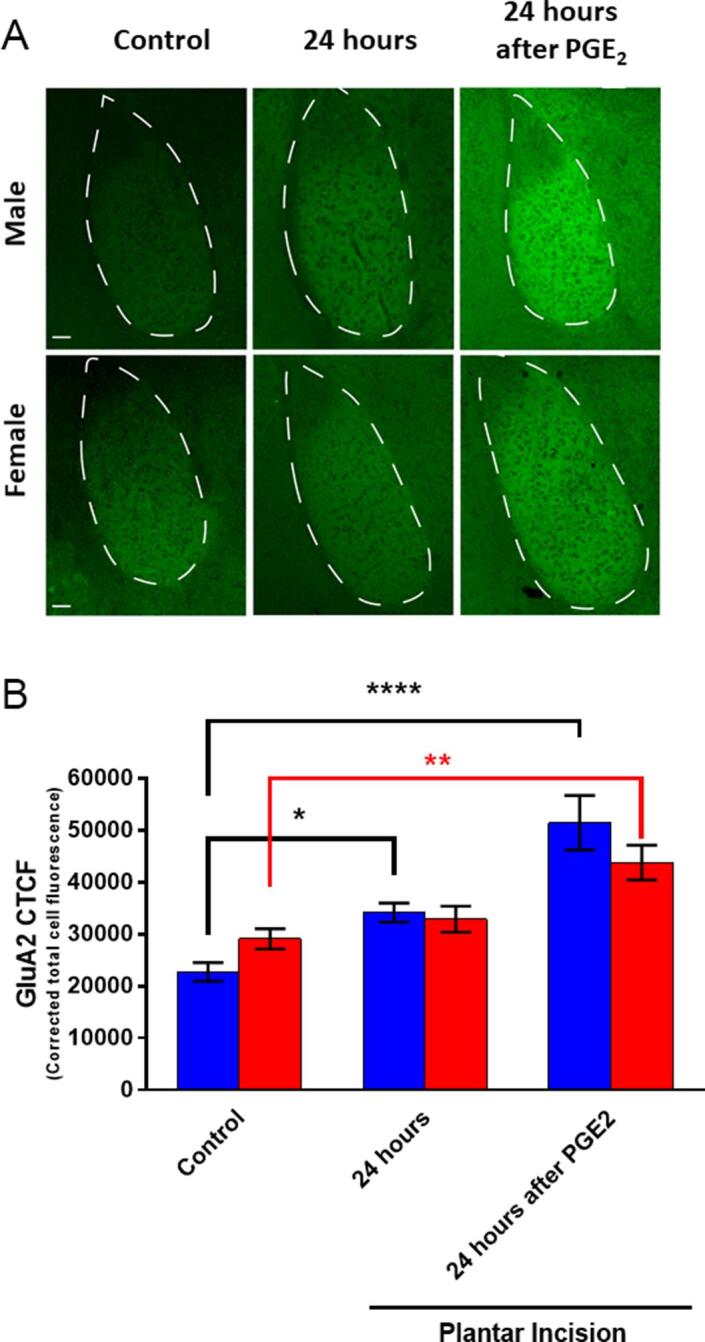
*Changes in GluA2 expression in BLA in hyperalgesic priming in male and female mice.***(A)** Representative 20X images showing GLUA2 immunoreactivity in female and male mice without incision (control), 24 hr after incision, and 24 hr after PGE_2_ injection in primed mice. **(B)** Corrected total cell fluorescence (CTCF) in males and females submitted to plantar incision (n = 4–5 group). Two-way ANOVA with Bonferroni *post hoc* test *p < 0.05; **p < 0.01; ****p < 0.0001. Black stars for male differences. Red stars and bar for female differences. Scale bar = 50 μm. (For interpretation of the references to colour in this figure legend, the reader is referred to the web version of this article.)

Finally, we examined GluA2 expression in the BLA 24 hr and after incision and 24 hr following the second stimulus, PGE_2_, in both male and female mice. GluA2 expression, measured by immunohistochemistry, was increased at both time points in male mice but only at the 24 hr following the second stimulus, PGE_2_, time point in female mice (Fig. 7A and B). GluA2 staining had a diffuse, neuropil staining pattern, consistent with dendritic localization for this ion channel. Unlike with PKCζ/PKMζ, where we only observed changes in male mice, GluA2 changes were induced by incision and/or by PGE_2_ injection in the BLA in both male and female mice. Our experimental design in does not allow a clear determination for the cause of this increase in female mice.

## 4 Discussion

Several previous studies have demonstrated a role for PKMζ in the spinal cord in the development and maintenance of chronic pain states (Asiedu et al., 2011, Laferriere et al., 2011, Melemedjian et al., 2013, Price and Ghosh, 2013, Nasir et al., 2016, Chen et al., 2018, George et al., 2019, Martin et al., 2019). The role of brain aPKCs in the regulation of chronic pain has not been studied as widely, except in the case of an important role of aPKCs in the ACC in neuropathic allodynia (Li et al., 2010, King et al., 2012). PKMζ, a prominently studied aPKC in the learning and memory field (Pastalkova et al., 2006, Shema et al., 2007, Migues et al., 2010, von Kraus et al., 2010, Sacktor, 2011, Sacktor and Hell, 2017), received a great deal of attention as a potential “memory molecule” (Sacktor, 2011); however, knockout studies have brought controversy to this area and it is now quite unclear what ZIP targets to influence late long-term potentiation (l-LTP) and long-term memory. It is likely that ZIP acts on targets that are unrelated to PKCζ/PKMζ, as has been shown in previous studies (Volk et al., 2013, LeBlancq et al., 2016) and since *Prkcz^−/−^* mice do not have a clear l-LTP or learning and memory phenotype, and ZIP is still active in these mice (Lee et al., 2013, Volk et al., 2013). Previous work from the Coderre lab demonstrates that *Prkcz^−/−^* mice do have a nociceptive phenotype, wherein they show deficits in the development of long-lasting hypersensitivity states in pain models, similar to the hyperalgesic priming paradigm (Nasir et al., 2016, George et al., 2019). Interestingly, this phenotype is only seen in male mice. Our findings using the NGF priming paradigm, albeit in a small cohort of mice, are consistent with those previous findings. This establishes PKCζ/PKMζ, which are both encoded by the *Prkcz* gene (Hernandez et al., 2003), as potential targets for the reversal of persistent pain states, but potentially only in males.

We focused the majority of our work on a potential role for the BLA in hyperalgesic priming. The BLA encodes inputs from many brain regions and is a key output area of the amygdala to the prefrontal cortex. The BLA contains an ensemble of neurons that are integrally involved in encoding the unpleasantness of pain (Corder et al., 2019) and elegant circuit-based studies are revealing how distinct subsets of BLA neurons regulate pro- and anti-nociceptive responses (Ji et al., 2010, Neugebauer, 2015, Huang et al., 2019). Given this critical role for the BLA in nociception, we hypothesized that manipulating molecular mechanisms of long-term synaptic plasticity in this brain region could have a strong impact on hyperalgesic priming. Our results provide evidence for our hypothesis, but only in the initiation of hyperalgesic priming. Bilateral injections of ZIP or pep2m were effective in blocking the establishment of hyperalgesic priming when they were given 24 hr after hindpaw incision. However, with pep2m, we did not observe any effect when this compound was given during the maintenance phase of hyperalgesic priming. This is different than observations when ZIP or pep2m are given intrathecally in the hyperalgesic priming or similar paradigms, in which case both peptides block the development of hyperalgesic priming and also reverse established priming in male rodents (Asiedu et al., 2011, Laferriere et al., 2011, Melemedjian et al., 2013, Nasir et al., 2016, George et al., 2019). ZIP is not effective in female rats, however, pep2m is efficacious when given intrathecally in both male and female rats (George et al., 2019). In our work in the BLA, ZIP and pep2m were effective in male and female mice for the prevention of hyperalgesic priming, as measured by mechanical hypersensitivity. ZIP did not have an effect on grimacing in male or female mice, but pep2m did have an effect in both sexes. Although ZIP has a behavioral effect in male and female mice, we only observed an increase in aPKC expression in the BLA in male mice. This latter finding is consistent with our findings in *Prkcz^−/−^* mice and with previous findings in rats from the Coderre group (Nasir et al., 2016, George et al., 2019). The effects of ZIP could be mediated by a non-specific effect, as has been proposed by others (Lee et al., 2013, Volk et al., 2013, LeBlancq et al., 2016).

We noted prominent differences in behavioral outcomes in the sucrose splash test in mice following PGE_2_ injection in primed animals. We observed that females and males receiving the scrambled peptide control and plantar incision showed a decrease in grooming compared to the naïve group (mice without plantar incision). These results demonstrate that hyperalgesic priming caused by plantar incision can induce behavioral responses, similar to those observed after chronic stress and reversed by classical antidepressant drugs (Yalcin et al., 2008, Boulle et al., 2014). To our knowledge, this is the first demonstration of changes in the sucrose splash test in hyperalgesic priming models. Intra-BLA microinjections of ZIP or pep2m around the time of incision blocked the development of this altered behavioral response in both males and females. With pep2m treatment, we tested whether this could be reversed by later treatment, just before the PGE_2_ stimulus. Consistent with our von Frey and grimace observations, late treatment with pep2m had no effect in either sex.

A primary proposed function of aPKCs in memory and synaptic plasticity is regulation of GluA2 trafficking (Migues et al., 2010, Sacktor, 2011). Our results with intra-BLA microinjections of pep2m, a peptide that interferes in the trafficking, cell surface expression and function of GluA2-containing AMPA receptors (Shi et al., 2001), demonstrated that this receptor is essential for induction of hyperalgesic priming independently of sex. Intra-BLA microinjection of pep2m strongly suppressed mechanical hypersensitivity in hyperalgesic priming and in affective responses after PGE_2_ injections in both males and females, suggesting that the role of this pathway in the BLA is not sex-dependent. Unlike with PKCζ/PKMζ immunoreactivity, we noted increased GluA2 in the BLA in both males and females, although the effect emerged more rapidly in male mice. Our findings with GluA2 are strikingly similar to observations made in the spinal cord using a similar model in rats (George et al., 2019). An important difference is that GluA2 seems to be critical for initiation and maintenance of priming-like effects in the spinal cord (Asiedu et al., 2011, George et al., 2019), but it is only involved in initiation in the BLA. A key area of study for future experiments will be to identify upstream regulation mechanisms of GluA2 in female rodents in pain models.

Our work points to a key role of BLA GluA2 in the initiation but not maintenance of hyperalgesic priming caused by plantar incision. Our findings also further corroborate a male sex specific role for PKMζ/PKCζ in the development and maintenance of chronic pain. A key message emerging from our experiments is that distinct, sex-dependent upstream mechanisms can converge on a single downstream event, in our case GluA2 in the BLA, to promote the development of nociceptive hypersensitivity. The fact that very similar observations have been made at the level of the spinal cord (George et al., 2019) demonstrate that these sex differences are present at different levels of the neuraxis. These collective findings advance our understanding of how molecular sex differences can converge to cause similar behavior phenotypes between sexes.

## Data availability statement

Raw data is available upon request to the corresponding author.

## Author contributions

DB, RN, AC, SG, CMF and TJP conceived of the project. DB, DT, SM, SS and IS carried out the experiments, data analysis, and drafting of the manuscript. SS, RN, AC and contributed to the formulation and supervision of the experiments and drafting the manuscript. SG provided the knockout mice for the study. TJP wrote the final manuscript.

## Funding

DB was a recipient of 10.13039/501100001807FAPESP research fellowship (2018/04775-0). AC and RN were recipient 10.13039/501100003593CNPq research (451500/2013-0 and 306556/2015-4, respectively).This work was supported by 10.13039/100000002NIH grant NS065926 and NS115441 to TJP.

## CRediT authorship contribution statement

**Daniela Baptista-de-Souza:** Conceptualization, Formal analysis, Funding acquisition, Investigation, Methodology, Writing - original draft, Writing - review & editing. **Diana Tavares-Ferreira:** Investigation, Methodology. **Salim Megat:** Investigation, Methodology. **Ishwarya Sankaranarayanan:** Investigation, Methodology. **Stephanie Shiers:** Investigation, Methodology. **Christopher M. Flores:** Conceptualization, Writing - review & editing. **Sourav Ghosh:** Conceptualization, Writing - review & editing. **Ricardo Luiz Nunes-de-Souza:** Conceptualization, Writing - review & editing. **Azair Canto-de-Souza:** Conceptualization, Writing - review & editing. **Theodore J. Price:** Conceptualization, Formal analysis, Funding acquisition, Methodology, Writing - original draft.

## Declaration of Competing Interest

The authors declare that they have no known competing financial interests or personal relationships that could have appeared to influence the work reported in this paper.
