# Supplementary material for: Sex differences in the role of atypical PKC within the basolateral nucleus of the amygdala in a mouse hyperalgesic priming model
Source: Neurobiol Pain. 2020 Jun 4;8:100049. doi: 10.1016/j.ynpai.2020.100049 (PMC7284072; doi:10.1016/j.ynpai.2020.100049)
Supplement: Supplementary data 1 [file mmc1.docx]

**Supplementary Materials**


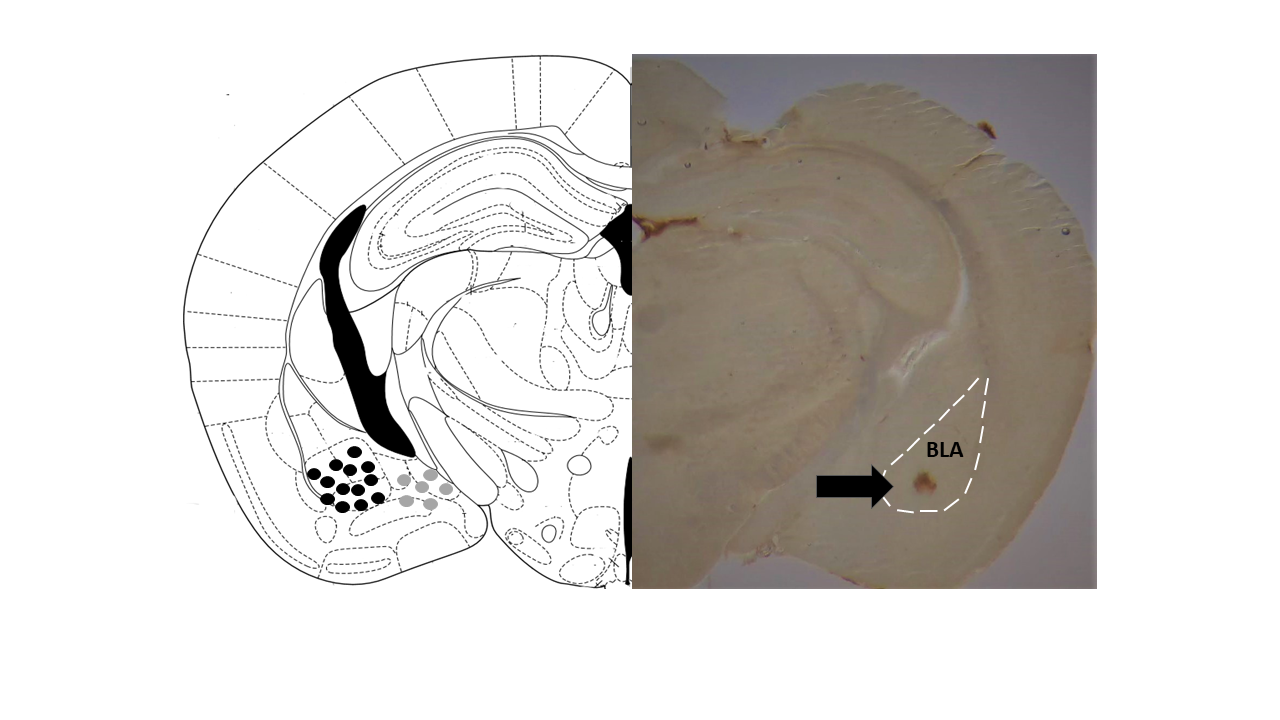


**Supplementary Figure 1.** Photomicrography of a coronal section of a representative subject showing an injection site within the mouse basolateral amygdaloid nucleus. Target sites for microinjection into the amygdala. Schematic representation of microinjection sites into the BLA. Black dots represent the sites of drug infusion that were on-target in the BLA, Gray dots represents sites of drug infusions were out-target in the BLA - Atlas of Paxinos and Franklin (2012).

**Supplementary Table 1** - Two-way ANOVA

|  |  | **Source of Variation**  **Genotype/Treatment/Sex** | | | **Time** | | | **Interaction** | | |
| --- | --- | --- | --- | --- | --- | --- | --- | --- | --- | --- |
| **Fig** | **Panel** | **dfn,dfd** | **F** | **p** | **dfn,dfd** | **F** | **p** | **dfn,dfd** | **F** | **p** |
| **1** | A | 1, 8 | 2.01 | 0.19 | 2.95, 23.63 | 23.22 | <0.0001 | 6, 48 | 0.59 | 0.72 |
|  | B | 1, 8 | 1.92 | 0.20 | 1.37,10.97 | 28,49 | 0.0001 | 2, 16 | 0.97 | 0.39 |
|  | C | 1, 5 | 0.01 | 0.90 | 2.61, 13.06 | 16.20 | 0.0002 | 6, 30 | 0.55 | 0.75 |
|  | D | 1, 5 | 0.03 | 0.86 | 1.17, 5.88 | 17,56 | 0.005 | 2, 10 | 0.09 | 0.90 |
|  | E | 1, 6 | 4,16 | 0.08 | 2.95, 17.71 | 14.34 | <0.0001 | 6, 36 | 0.42 | 0.86 |
|  | F | 1, 6 | 3.53 | 0.10 | 1.39, 8.36 | 14.94 | 0.0028 | 2, 12 | 1,20 | 0.33 |
|  |  |  |  |  |  |  |  |  |  |  |
| **2** | A | 1,15 | 4.85 | 0.04 | 3.43, 51.54 | 63.60 | <0.0001 | 5, 75 | 3.88 | 0.003 |
|  | B | 1,15 | 0.01 | 0.90 | 2.99, 44.86 | 46.68 | <0.0001 | 5, 75 | 1.33 | 0.25 |
|  | C | 1,11 | 12.24 | 0.005 | 3.20, 35.28 | 46.67 | <0.0001 | 5, 55 | 9.63 | <0.0001 |
|  | D | 1,11 | 4.97 | 0.04 | 2.89, 31.81 | 44.43 | <0.0001 | 5, 55 | 1.57 | 0.18 |
|  |  |  |  |  |  |  |  |  |  |  |
| **4** | B | 1, 26 | 119.7 | <0.0001 | 3, 26 | 37.28 | <0.0001 | 3, 26 | 32.14 | <0.0001 |
|  |  |  |  |  |  |  |  |  |  |  |
| **5** | A | 2,18 | 9.60 | 0.0015 | 4.32, 77.84 | 47.24 | <0.0001 | 10, 90 | 2.75 | 0.0052 |
|  | B | 2,16 | 3.31 | 0.062 | 3.04, 48.74 | 77.45 | <0.0001 | 10, 80 | 2.64 | 0.0078 |
|  | C | 2,15 | 10.22 | 0.0016 | 3.71, 55.72 | 30.18 | <0.0001 | 10, 75 | 1.79 | 0.0754 |
|  | D | 2,15 | 29.39 | <0.0001 | 2.95, 44.36 | 50.34 | <0.0001 | 10, 75 | 3.69 | 0.0005 |
|  |  |  |  |  |  |  |  |  |  |  |
| **7** | B | 1,19 | 0.123 | 0.7292 | 2, 19 | 26.59 | <0.0001 | 2, 19 | 2.70 | 0.0092 |
|  |  |  |  |  |  |  |  |  |  |  |

**Supplementary Table 2** - Two-way ANOVA Bonferroni comparisons

| **Bonferroni** | | | | | |
| --- | --- | --- | --- | --- | --- |
| Fig | Comparison | p | Fig | Comparison | p |
| **1A** | **Response** |  |  | **Response** |  |
|  | WT- NGF (50 ng) - PKCz KO NGF (50 ng) |  | **2C** | Scrambled ZIP - ZIP |  |
|  | BL | >0,9999 |  | Baseline | >0,9999 |
|  | 0.2 | 0,7914 |  | 24h | >0,9999 |
|  | 1 | 0,9731 |  | 72h | 0,8370 |
|  | 2 | 0,4061 |  | 10d | >0,9999 |
|  | 3 | >0,9999 |  | 3h | 0,0339 |
|  | 4 | >0,9999 |  | 24h | >0.0001 |
|  | 8 | >0,9999 |  |  |  |
|  |  |  | **2D** | Scramble - ZIP |  |
| **1B** | WT- NGF (50 ng) - PKCz KO NGF (50 ng) |  |  | Baseline | >0,9999 |
|  | Day 8 BL | >0,9999 |  | 3h | >0,9999 |
|  | 3h | 0,2006 |  | 24h | >0,9999 |
|  | 24h | 0,2625 |  | 48h | >0,9999 |
|  |  |  |  | 3h | 0,6734 |
| **1C** | WT- NGF (50 ng) - PKCz KO NGF (50 ng) |  |  | 24h | 0,2342 |
|  | BL | >0,9999 |  |  |  |
|  | 0.2 | >0,9999 | **4B** | Males |  |
|  | 1 | >0,9999 |  | Control vs. 24 hours | >0,9999 |
|  | 2 | 0,2546 |  | Control vs. 2 weeks | <0,0001 |
|  | 3 | >0,9999 |  | Control vs. Priming | <0,0001 |
|  | 4 | >0,9999 |  | Females |  |
|  | 8 | >0,9999 |  | Control vs. 24 hours | 0,1127 |
|  |  |  |  | Control vs. 2 weeks | 0,3585 |
| **1D** | WT- NGF (50 ng) - PKCz KO NGF (50 ng) |  |  | Control vs. Priming | 0,6260 |
|  | Day 8 BL | >0,9999 |  |  |  |
|  | 3h | 0,1709 | **5A** | Baseline |  |
|  | 24h | >0,9999 |  | Scramble vs. pep2m - Initiation | 0,4135 |
|  |  |  |  | Scramble vs. pep2m - Priming | >0,9999 |
| **1E** | WT- NGF (50 ng) - PKCz KO NGF (50 ng) |  |  | 1d |  |
|  | BL | >0,9999 |  | Scramble vs. pep2m - Initiation | 0,0487 |
|  | 0.2 | 0,5022 |  | Scramble vs. pep2m - Priming | 0,4719 |
|  | 1 | >0,9999 |  | 3d |  |
|  | 2 | >0,9999 |  | Scramble vs. pep2m - Initiation | 0,0482 |
|  | 3 | 0,3903 |  | Scramble vs. pep2m - Priming | >0,9999 |
|  | 4 | >0,9999 |  | 14d |  |
|  | 8 | >0,9999 |  | Scramble vs. pep2m - Initiation | 0,1118 |
|  |  |  |  | Scramble vs. pep2m - Priming | >0,9999 |
| **1F** | WT- NGF (50 ng) - PKCz KO NGF (50 ng) |  |  | 3h |  |
|  | Day 8 | 1.05 |  | Scramble vs. pep2m - Initiation | 0,0237 |
|  | 3h | 0.59 |  | Scramble vs. pep2m - Priming | >0,9999 |
|  | 24h | 0.02 |  | 24h |  |
|  |  |  |  | Scramble vs. pep2m - Initiation | 0,0010 |
| **2A** | ZIP Scramble - ZIP |  |  | Scramble vs. pep2m - Priming | 0,3564 |
|  | Baseline | >0,9999 |  |  |  |
|  | 24h | >0,9999 | **5B** | Baseline |  |
|  | 72h | 0,4818 |  | Scramble vs. pep2m | 0,6701 |
|  | 14 days | >0,9999 |  | Scramble vs. pep2m - Priming | 0,3062 |
|  | 3 horas | 0,2339 |  | 3h |  |
|  | 24horas | 0,0019 |  | Scramble vs. pep2m | 0,6731 |
|  |  |  |  | Scramble vs. pep2m - Priming | 0,3176 |
| **2B** | Scramble - ZIP |  |  | 24h |  |
|  | Baseline | >0,9999 |  | Scramble vs. pep2m | >0,9999 |
|  | 3h | >0,9999 |  | Scramble vs. pep2m - Priming | >0,9999 |
|  | 24h | >0,9999 |  | 48h |  |
|  | 48h | >0,9999 |  | Scramble vs. pep2m | >0,9999 |
|  | 3h | >0,9999 |  | Scramble vs. pep2m - Priming | >0,9999 |
|  |  |  |  | 3h |  |
|  |  |  |  | Scramble vs. pep2m | 0,0465 |
|  |  |  |  | Scramble vs. pep2m - Priming | >0,9999 |
|  |  |  |  | 24h |  |
|  |  |  |  | Scramble vs. pep2m | 0,0397 |
|  |  |  |  | Scramble vs. pep2m - Priming | 0,8474 |
| **Bonferroni** | | | | | |
| Fig | Comparison | p | Fig | Comparison | p |
|  | **Response** |  |  | **Response** |  |
| **5C** | Baseline |  | **7B** | Males |  |
|  | Scramble vs. pep2m - Initiation | >0,9999 |  | Control vs. 24 hours | 0,0217 |
|  | Scramble vs. pep2m - Priming | 0,8984 |  | Control vs. 14 days | <0,0001 |
|  | 1d |  |  | Females |  |
|  | Scramble vs. pep2m - Initiation | 0,0938 |  | Control vs. 24 hours | 0,7704 |
|  | Scramble vs. pep2m - Priming | 0,5553 |  | Control vs. 14 days | 0,0055 |
|  | 3d |  |  |  |  |
|  | Scramble vs. pep2m - Initiation | 0,6754 |  |  |  |
|  | Scramble vs. pep2m - Priming | 0,8598 |  |  |  |
|  | 10d |  |  |  |  |
|  | Scramble vs. pep2m - Initiation | 0,0582 |  |  |  |
|  | Scramble vs. pep2m - Priming | 0,5914 |  |  |  |
|  | 3h |  |  |  |  |
|  | Scramble vs. pep2m - Initiation | 0,0527 |  |  |  |
|  | Scramble vs. pep2m - Priming | >0,9999 |  |  |  |
|  | 24h |  |  |  |  |
|  | Scramble vs. pep2m - Initiation | 0,0149 |  |  |  |
|  | Scramble vs. pep2m - Priming | 0,6580 |  |  |  |
|  |  |  |  |  |  |
| **5D** | Baseline |  |  |  |  |
|  | Scramble vs. pep2m | >0,9999 |  |  |  |
|  | Scramble vs. pep2m - Priming | >0,9999 |  |  |  |
|  | 3h |  |  |  |  |
|  | Scramble vs. pep2m | 0,0214 |  |  |  |
|  | Scramble vs. pep2m - Priming | 0,6612 |  |  |  |
|  | 24h |  |  |  |  |
|  | Scramble vs. pep2m | 0,0032 |  |  |  |
|  | Scramble vs. pep2m - Priming | >0,9999 |  |  |  |
|  | 48h |  |  |  |  |
|  | Scramble vs. pep2m | >0,9999 |  |  |  |
|  | Scramble vs. pep2m - Priming | 0,9014 |  |  |  |
|  | 3h |  |  |  |  |
|  | Scramble vs. pep2m | 0,0614 |  |  |  |
|  | Scramble vs. pep2m - Priming | 0,8502 |  |  |  |
|  | 24h |  |  |  |  |
|  | Scramble vs. pep2m | 0,0008 |  |  |  |
|  | Scramble vs. pep2m - Priming | 0,6968 |  |  |  |

**Supplementary Table 3** - One-way ANOVA with Bonferroni comparisons

| **One-way ANOVA** | | | | | | | | |
| --- | --- | --- | --- | --- | --- | --- | --- | --- |
|  | | **Between treatment** | **Within treatment** |  | | |  | **Bonferroni** |
| **Fig** | **Panel** | **MS** | **MS** | **dfn,dfd** | **F** | **p** | **Comparison** | **p** |
| **3** | **A** | 187.3 | 27.06 | 2, 20 | 6.92 | 0.0052 | Naive vs. Scrambled ZIP | 0,0047 |
|  |  |  |  |  |  |  | Naive vs. ZIP | 0,0860 |
|  |  |  |  |  |  |  | Scrambled ZIP vs. ZIP | >0,9999 |
|  | **B** | 42.27 | 34.78 | 2, 15 | 1.21 | 0.3243 | Naive vs. Scrambled ZIP | 0,4197 |
|  |  |  |  |  |  |  | Naive vs. ZIP | >0,9999 |
|  |  |  |  |  |  |  | Scrambled ZIP vs. ZIP | >0,9999 |
|  | **C** | 401.3 | 75.56 | 2, 21 | 5.31 | 0.0136 | Naive vs. Scrambled ZIP | 0,0353 |
|  |  |  |  |  |  |  | Naive vs. ZIP | >0,9999 |
|  |  |  |  |  |  |  | Scrambled ZIP vs. ZIP | 0,0393 |
|  | **D** | 1765 | 250.8 | 2,17 | 7.03 | 0.0059 | Naive vs. Scrambled ZIP | 0,0565 |
|  |  |  |  |  |  |  | Naive vs. ZIP | 0,4420 |
|  |  |  |  |  |  |  | Scrambled ZIP vs. ZIP | 0,0048 |
|  |  |  |  |  |  |  |  |  |
| **6** | **A** | 315.2 | 18.19 | 2, 18 | 17.33 | <0.0001 | Scrambled peptdie vs. pep2m - Initiation | **** |
|  |  |  |  |  |  |  | Scrambled peptdie vs. pep2m - Priming | * |
|  |  |  |  |  |  |  | pep2m - Initiation vs. pep2m - Priming | ** |
|  | **B** | 309.1 | 13.82 | 2, 14 | 22.36 | <0.0001 | Scrambled peptdie vs. pep2m - Initiation | <0,0001 |
|  |  |  |  |  |  |  | Scrambled peptdie vs. pep2m - Priming | 0,0104 |
|  |  |  |  |  |  |  | pep2m - Initiation vs. pep2m - Priming | 0,0380 |
|  |  |  |  |  |  |  |  |  |
|  | **C** | 5867 | 167.6 | 2, 18 | 35.01 | <0.0001 | Scrambled peptdie vs. pep2m - Initiation | **** |
|  |  |  |  |  |  |  | Scrambled peptdie vs. pep2m - Priming | * |
|  |  |  |  |  |  |  | pep2m - Initiation vs. pep2m - Priming | **** |
|  | **D** | 2714 | 625.2 | 2, 14 | 4.34 | 0.00341 | Scrambled peptdie vs. pep2m - Initiation | 0,0328 |
|  |  |  |  |  |  |  | Scrambled peptdie vs. pep2m - Priming | 0,8118 |
|  |  |  |  |  |  |  | pep2m - Initiation vs. pep2m - Priming | 0,3642 |
